# EPMDA: an expression-profile based computational model for microRNA-disease association prediction

**DOI:** 10.18632/oncotarget.18788

**Published:** 2017-06-28

**Authors:** Yu-An Huang, Zhu-Hong You, Li-Ping Li, Zhi-An Huang, Lu-Xuan Xiang, Xiao-Fang Li, Lin-Tao Lv

**Affiliations:** ^1^ College of Information Engineering, Xijing University, Xi’an 710123, China; ^2^ Xinjiang Technical Institute of Physics and Chemistry, Chinese Academy of Science, Urumqi 830011, China; ^3^ College of Computer Science and Software Engineering, Shenzhen University, Shenzhen, 518060, China; ^4^ Institute of Information and Control, Hangzhou Dianzi University, Hangzhou, China

**Keywords:** disease, MicroRNA, expression profile, biomarker

## Abstract

MicroRNA has become a new star molecule for understanding multiple biological processes and the mechanism of various complex human diseases. Even though a number of computational models have been proposed for predicting the association between microRNAs and various human diseases, most of them are mainly based on microRNA functional similarity and heterogeneous biological networks which suffer from inevitable computational error and bias. In this work, considering the limitation of information resource used by existing methods, we proposed EPMDA model which is the first computational method using the expression profiles of microRNAs to predict the most potential microRNAs associated with various diseases. Based on the dataset constructed from HMDD v2.0 database, EPMDA obtained AUCs of 0.8945 and 0.8917 based on the leave-one-out and 5-fold cross validation, respectively. Furthermore, EPMDA was applied to two important human diseases. As a result, 80% and 88% microRNAs in the top-25 lists of Colon Neoplasms and Kidney Neoplasms were confirmed by other databases. The performance comparison of EPMDA with existing prediction models and classical algorithms also demonstrated the reliable prediction ability of EPMDA. It is anticipated that EPMDA can be used as an effective computational tool for future biomedical researches.

## INTRODUCTION

MicroRNAs (abbreviated miRNAs) are a kind of small non-coding RNA molecule which contains ~22 nucleotides and can be found in plants, animals and some viruses [1, 2]. As a breakthrough medical discovery, microRNA has been found to get involved in various biological processes [3, 4]. Specifically, it can cause degradation and repression of RNA transcripts through complete or partial sequence complementarity, and further negatively regulates gene expression at the levels of messenger RNAs (mRNAs) [5, 6]. Even though the majority of biological functions are directly carried out by the proteins which are coded by the protein-coding genes, these genes only take up an extremely minority of the human genome (approximately 1.5%). Besides, according to the report of international Encyclopedia of DNA Elements (ENCODE) project, it is shown that at least 80% of human genomic DNA has biochemical activity [7, 8]. Therefore, microRNAs, along with other kinds of noncoding RNA (i.e. lncRNA, circularRNA and snoRNA), has been considered as an important supplement for higher level of complexity and subtlety in human gene function.

The study of microRNAs has obtained a big progress for the past decade since the first microRNA, lin-4, and the second microRNA, let-7, were discovered in 1993 and 2000 [9, 10]. So far, the amount of identified mature microRNAs has been achieved to 2588 according the latest version of miRBase database [6]. Previous biological studies and researches offer great insights into the complex mechanism of microRNA functions, which is engaged by various microRNA-target interactions. Accumulating evidences have shown that various kinds of biological molecules can interact with microRNAs which function in RNA silencing, and therefore further influence the post-transcriptional regulation of gene expression. Recently, the novel hypothesis of CeRNA (competing endogenous RNA) gives a new explanation for how other RNA transcripts interact with microRNAs [11, 12]. According to this hypothesis, the pool of transcribed psedogenes, lncRNAs, circRNAs compete to cooperatively sequester microRNAs through microRNA response elements (MREs). Specially, the ceRNA network of PTEN, which is a critical tumor suppressor gene, has been systematically studied. CNOT6L, VAPA and ZEB2 have been identified as ceRNAs to regulate PTEN expression level in a microRNA-dependent manner [13–15].

Along with the progress of molecular biology study, increasing researches show that microRNAs can carry out essential functions in various biological processes including metabolism[16, 17], differentiation [18], proliferation [19], signal transduction [20] and apoptosis [21]. However, for the majority of identified microRNAs, their biological functions are still unclear partially due to the extreme complexity of microRNA regulation networks. Even though the specific regulation mechanism of most microRNAs is still unclear, increasing evidences have shown that the expression level of microRNAs has a close relationship with the development of diverse human diseases. Therefore, it is feasible to regard microRNAs as biomarkers to help to understand the underlying molecular and pathological mechanisms of complex human diseases. The relationship between microRNAs and disease incidence has been widely studied. For examples, miR-195 with higher expression level was found to reduce breast tumor cell survival and increase apoptosis by downregulating the expression of Raf-1, Bcl-2, and P-glycoprotein [22]. The overexpression of miR-145 was confirmed to inhibit the proliferation of transfected lung adenocarcinoma cell through the downregulation of mRNA expression of EGFR and NUDT1 [23]. Furthermore, overly-expressed miR-135a and miR-335 were shown to contribute to the progression of colorectal cancer [24]. Discovering disease-associated microRNAs can not only lead to new approaches for disease diagnosis, treatment and prevention at the molecular level but can also discover effective molecular targets for the drug development.

However, the mainstream experiment-based approaches for microRNA-disease interaction identification are still inefficient since the identification work usually needs to collect sufficient clinical data which need time and money. Even though some biomarkers for specific diseases have been confirmed, the function mechanism of microRNAs in most complex diseases is still unclear yet. Therefore, as an important supplement for biological experiments, computational model-based methods for microRNA-disease association have been attracting increasing attention from researches in this field. Specially, some important databases storing experimentally-confirmed microRNA-disease associations have been built, which provides essential data resources for extensive studies in this field [25–27].

So far, there are several computational models have been proposed for inferring new microRNA-disease associations, which can be mainly classified into three categories. The first category is mainly based on network similarity measurement. For example, Xuan *et al*. have proposed the model of MIDP which is mainly based on the assumption that functionally similar microRNAs tend to be involved in similar diseases [28]. Specifically, MIDP model constructs a microRNA functional similarity network (i.e. Mnet) by measuring the semantic similarities of diseases. The second category uses machine learning algorithms to predict the most potential microRNA associated with specific diseases. For examples, Xu *et al*. have develop a supervised learning-based classification model which constructs 4-dimension vectors to represent microRNA features and then applies support vector machine to predict whether a query microRNA is associated with specific disease or not [29]. The final category tries to combine different extra biological information for predicting microRNA-disease association, which are motivated by the consideration that the amount of experimentally-confirmed microRNA-disease association data are still far from sufficient for training. For example, Mørk *et al*. have developed the model of miRPD which is mainly based on a constructed microRNA-protein-disease heterogeneous association network [30]. Even though most of known microRNA-disease associations are confirmed by detecting the change of expression level of microRNA along with different disease development statuses, there has been no computational model considering the information of microRNA expression distribution in human tissues.

Accumulating evidences show that the deregulation of microRNAs can usually cause diverse human diseases since microRNAs expression pattern can play a significant role in chromatin dynamics and gene silencing [31–33]. Therefore, the information of microRNA expression can offer important insights into the relationship between microRNAs and diseases. In this work, we proposed a novel computational model called EPMDA for inferring microRNA-disease associations, which is mainly based on the assumption that microRNAs sharing similar distribution in human tissues are more possible to get involved in similar diseases. Specifically, we first used the expression profile data to calculate the similarity scores between microRNAs. Following Li's work, EPMDA model constructs a disease similarity matrix by introducing the semantic similarity [34]. Finally, we further adopted a two-way diffusion approach to calculate the association possibility of the unknown microRNA-disease association. To evaluate the performance of EPMDA model, we have adopted two cross validation frameworks of leave-one-out cross validation (LOOCV) and 5-fold cross validation (5-fold CV) to use the proposed method to predict the most potential microRNA-disease associations based on the HMDD v2.0 database [26]. Furthermore, we have also analyzed the predicted microRNA lists of two kinds of important diseases. The ROC curves (receiver-operating characteristic curves) and AUC (area under ROC curve) values were calculated for each experiments. As a result, the EPMDA model yielded AUCs of 0.8945 and 0.8914+/−0.0004 based on LOOCV and 5-fold cross validation, respectively. By comparing the previously-proposed prediction models and the proposed method, the outstanding performance demonstrated the effective prediction ability of EPMDA model. It is anticipated that EPMDA can be applied to be used to predict the most potential microRNA-disease associations on a large scale, which facilitates future disease biomarker discovery and new drug development.

## RESULTS

### Performance evaluation

To evaluate the performance of EPMDA model, we implemented the method of LOOCV to predict the microRNA-disease association possibility based on the dataset downloaded from HMDD v2.0 database. Specifically, each known microRNA-disease association was left out in turn as a test sample and the other known microRNA-disease associations were used for training. For each testing round, the test sample obtaining higher ranks than the given threshold would be considered as a successful prediction while those with ranks lower than the threshold was regarded as unsuccessful predictions. We calculate the corresponding true positive rates (TPR, sensitivity) and false positive rates (FPR, 1-specificity) by setting different thresholds. The ROC curves for each experiment were computed by plotting TPR versus FPR at different thresholds. The values of area under ROC curve were also computed. AUC of 0.5 means a purely random prediction and a higher AUC value means a better prediction result.

**Figure 1:**
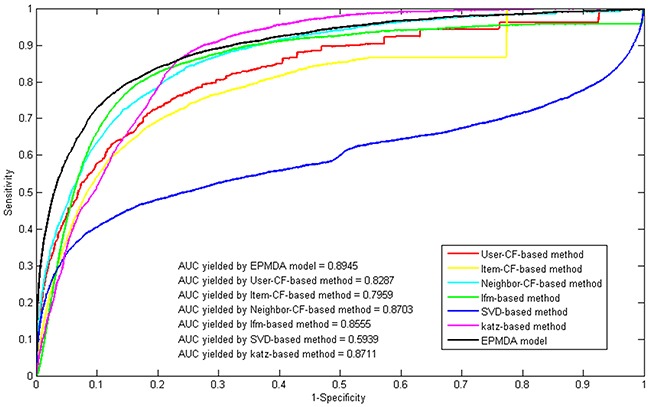
Performance comparison between EPMDA and six other classical prediction models in terms of ROC curves and AUCs based on leave-one-out cross validation As a result, EPMDA yielded the best performance with the highest AUC of 0.8945.

Furthermore, we compared the performance of EPMDA with some classical recommended algorithms (i.e., user-based collaborative filtering, item-based collaborative filtering, neighbor-based collaborative filtering, latent factor model, svd-based model) and social network prediction algorithm (i.e., Katz-based method) [35]. Since the task of microRNA-disease association prediction can be regarded as a matrix filling problem, we can obtain the most potential microRNA-disease association through applying the collaborative filtering methods on the adjacency matrix constructed by the known microRNA-disease associations. User-based and item-based CF are two basic memory-based recommendation algorithms which respectively computes the average ratings for each item rated by similar users and the average ratings for each user rated by similar items. And neighbor-based CF is an integrated version of user-based and item-based CF and takes the weighted average of all the ratings of these two CFs. Similarly, we can also regard the known microRNA-disease association network as a classical social network and implement the social network prediction model on it. Specifically, Katz method which was previously used to predict microbe-disease and lncRNA-disease associations was also explored in this work [35, 36]. In this series of comparison experiments, all algorithms were implemented by introducing the same inputs (i.e., microRNA expression similarity matrix and disease semantic similarity matrix). As a result, the proposed model of EPMDA yielded the best performance among all method, with the highest AUC of 0.8945 while the rest methods (i.e. user-based collaborative filtering, item-based collaborative filtering, neighbor-based collaborative filtering, latent factor model, svd-based model and Katz-based method) yielded poorer prediction performance with AUCs of 0.8287, 0.7959, 0.8703, 0.8555, 0.5939 and 0.8711, respectively (see Figure 1).

Furthermore, 5-fold cross validation was also adopted for evaluating the prediction performance of EPMDA. All known microRNA-disease associations would be first randomly separated into 5 groups of roughly same size. In each round of 5-fold cross validation, 4 groups of samples were used for training while the rest one was used as testing samples. We further plotted the ROC curve and computed corresponding AUC value for each round. To avoid the bias of random division, we repeated the 5-fold cross validation for 20 times and computed the average AUC values as the final evaluation values for prediction performance. Similar with LOOCV experiments, we implemented six other classical methods and EPMDA model to predict the microRNA-disease associations based on HMDD database. As a result, we obtained the best prediction performance by using the EPMDA model with the high average AUC of 0.8914+/−0.0004 (see Table 1). User-based collaborative filtering, item-based collaborative filtering, neighbor-based collaborative filtering, latent factor model, svd-based model and Katz-based method yielded poorer prediction results with average AUCs of 0.8250, 0.7900, 0.8664, 0.8546, 0.5079 and 0.8570, respectively.

**Table 1:** Performance comparisons between EPMDA and six other classical recommendation algorithms and graph-based prediction model in term of average AUC based on 5-fold cross validation

METHOD	AVERAGE AUC
Used-based collaborative filtering method	0.8250+/−0.0007
Item-based collaborative filtering method	0.7900+/−0.0010
Neighbor-based collaborative filtering method	0.8664+/−0.0005
Latent factor model	0.8546+/−0.0004
SVD-based method	0.5079+/−0.0013
Katz-based social network prediction model	0.8570+/−0.0003
EPMDA model	0.8917+/−0.0004

**Figure 2:**
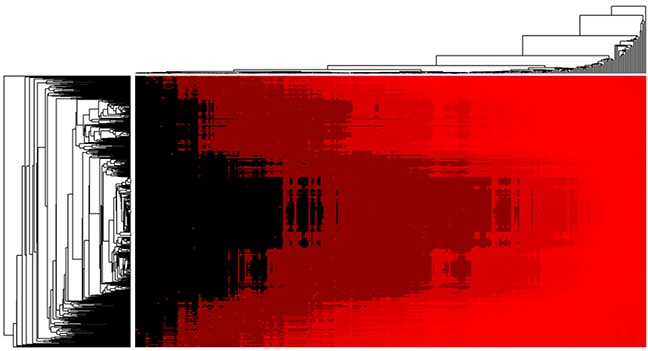
Heat map of microRNA-disease association possibility predicted by EPMDA in which rows and column denote microRNAs and diseases

So far, there have been some computational models proposed for predicting microRNA-disease associations. Some of them were performed by using the data of HMDD v2.0, which is the same data resource we explored in this work [34, 37–42]. Therefore, we simply compared the prediction performance of these methods. Most of models previously proposed make prediction by introducing the microRNA-microRNA functional similarity scores which can be downloaded from Wang's work [43]. However, the biological functions of most of microRNAs have not been well studied yet, and therefore it is inevitable to cause prediction bias if we simply introduce the computed function similarity of microRNAs. Instead of following Wang's previous work [43], we introduced a new kind of data, microRNA expression similarity, which is yielded by direct biological experiments and therefore can lead to less error. As can be seen from Table 2, the model of EPMDA yielded the highest prediction performance with the highest AUC of 0.8945 in LOOCV experiment and average AUC of 0.8917+/−0.0004 in 5-fold cross validation experiments. The compared methods of RLSMDA, HDMP, WBSMDA, MCMDA, HGIMDA, RWRMDA and RBMMMDA yielded smaller AUCs of 0.8426, 0.8366, 0.8030, 0.8749, 0.8781, 0.8617 and 0.8606 in terms of LOOCV. Furthermore, we further publicly released the rank list of microRNA-disease associations which was yielded by EPMDA based the dataset of HMDD (see Supplementary Table S1). The heat map of all the final prediction result is shown as Figure 2. It is anticipated that those microRNA-disease association obtaining high ranks will be verified by further research and experimental validtation.

**Table 2:** Performance comparisons between EPMDA and seven existing computational models (RLSMDA, HMDP, WBSMDA, MCMDA, HGIMDA RWRMDA and RBMMMDA) for predicting microRNA-disease association in terms of AUCs based on leave-one-out and 5-fold cross validations. All the eight models adopt the disease semantic similarity based on disease MeSH annotations

METHOD	LOOCV	5-fold cross validation
RLSMDA[38]	0.8426	0.6953
HDMP[42]	0.8366	0.7702
WBSMDA[37]	0.8030	0.8031
MCMDA[34]	0.8749	0.8767
HGIMDA[41]	0.8781	0.8077
RWRMDA[39]	0.8617	0.7891
RBMMMDA[40]	0.8606	N/A
EPMDA (The proposed method)	0.8945	0.8917+/−0.0004

### Case studies

In this section, to evaluate the effectiveness of our proposed method, we further analyzed the prediction results of two important diseases (i.e., colon neoplasms and kidney neoplasms). Specifically, we focused on the microRNAs which obtained the top25 ranks for the diseases of colon and kidney neoplasms and verified their accuracy by checking two other databases, miRNA2Disease and dbDEMC.

### Colon Neoplasms

Colon Neoplasms has come to be one of the deadliest threats to human life in all over the world. It is reported that around 50% patients of Colon Neoplasms cannot survive more than five years from first diagnosis due to the metastatic diseases [44, 45]. So far, there are some microRNAs having been identified to be associated with the development of Colon Neoplasms, and some of them could be regarded as the biomarkers for the early diagnosis and prevention. As can be seen from the Table 3, 80% of the microRNAs in the top 25 prediction list yielded by EPMDA model could be verified by the miR2Disease and dbDECMC database [25, 27]. Five microRNAs (i.e., hsa-mir-499a, hsa-mir-150, hsa-mir-208b, hsa-mir-103a and hsa-mir-151a) which obtained 3^rd^, 10^th^, 19^th^, 20^th^ and 23^th^ ranks were failed to be confirmed.

**Table 3:** Prediction results of microRNAs associated with Colon Neoplasms in top-25 ranking list

Rank	microRNA	Evidence	Rank	microRNA	Evidence
1	hsa-mir-125a	miR2Disease	14	hsa-mir-1	miR2Disease dbDEMC
2	hsa-mir-196a	miR2Disease	15	hsa-mir-133a	miR2Disease dbDEMC
3	hsa-mir-499a	**Unconfirmed**	16	hsa-mir-133b	miR2Disease dbDEMC
4	hsa-mir-198	dbDEMC	17	hsa-mir-146a	dbDEMC
5	hsa-mir-29a	miR2Disease dbDEMC	18	hsa-mir-155	miR2Disease dbDEMC
6	hsa-mir-29b	miR2Disease dbDEMC	19	hsa-mir-208b	**Unconfirmed**
7	hsa-let-7a	miR2Disease dbDEMC	20	hsa-mir-103a	**Unconfirmed**
8	hsa-mir-141	miR2Disease dbDEMC	21	hsa-mir-10b	miR2Disease dbDEMC
9	hsa-mir-143	miR2Disease dbDEMC	22	hsa-mir-135a	dbDEMC
10	hsa-mir-150	**Unconfirmed**	23	hsa-mir-151a	**Unconfirmed**
11	hsa-mir-15a	dbDEMC	24	hsa-mir-152	dbDEMC
12	hsa-mir-16	dbDEMC	25	hsa-mir-181b	miR2Disease dbDEMC
13	hsa-mir-21	miR2Disease dbDEMC			

### Kidney Neoplasms

Kidney Neoplasms is another common cause leading to death, which is usually accompanied by high rates of metastatic recurrences (~30% of localized renal cell carcinoma cases) and death (5-year survival rate of 60-70%) [46]. It is reported that there are more than 250,000 new cases diagnosed as Kidney Neoplasms with more than 40% mortality in the current years [47]. Along with the development of high-throughput sequencing technologies, researchers have discovered some microRNAs which are associated with the development and progression of Kidney Neoplasms. For example, miR-1233 has been identified as a potential biomarker for renal cell carcinoma (RCC) by using the technique of TaqMan Low Density Array and confirmed to be highly expressed in RCC patients by using quantitative real-time PCR [48]. Table 4 shows the top 25 prediction results for microRNA candidates associated with Kidney Neoplasms. There 88% microRNAs in the list have been confirmed by miRNA2Disease and dbDECMC database. The three unfirmed microRNAs (i.e., hsa-mir-208b, hsa-mir-103a and hsa-mir-151a) obtained respectively low ranks (i.e., 19^th^, 20^th^ and 25^th^), compared with the confirmed ones.

**Table 4:** Prediction results of microRNAs associated with Kidney Neoplasms in top-25 ranking list

Rank	microRNA	Evidence	Rank	microRNA	Evidence
1	hsa-mir-125a	miR2Disease dbDEMC	14	hsa-mir-1	dbDEMC
2	hsa-mir-196a	dbDEMC	15	hsa-mir-133a	dbDEMC
3	hsa-mir-499a	miR2Disease	16	hsa-mir-133b	dbDEMC
4	hsa-mir-198	miR2Disease dbDEMC	17	hsa-mir-146a	miR2Disease
5	hsa-mir-29a	miR2Disease dbDEMC	18	hsa-mir-155	dbDEMC
6	hsa-mir-29b	miR2Disease dbDEMC	19	hsa-mir-208b	**Unconfirmed**
7	hsa-let-7a	miR2Disease dbDEMC	20	hsa-mir-103a	**Unconfirmed**
8	hsa-mir-141	miR2Disease	21	hsa-mir-106a	miR2Disease dbDEMC
9	hsa-mir-143	miR2Disease dbDEMC	22	hsa-mir-10b	miR2Disease dbDEMC
10	hsa-mir-150	dbDEMC	23	hsa-mir-126	miR2Disease dbDEMC
11	hsa-mir-15a	miR2Disease dbDEMC	24	hsa-mir-135a	dbDEMC
12	hsa-mir-16	miR2Disease dbDEMC	25	hsa-mir-151a	**Unconfirmed**
13	hsa-mir-21	miR2Disease dbDEMC			

## DISCUSSION

In the past several years, microRNA has come to be a new star molecule in the studies on disease mechanism and bioinformatics and there are more and more researches focusing on using computational methods to predict novel microRNA-disease associations. EPMDA is a computational model for inferring the most potential microRNA biomarkers for specific human diseases by using the experimentally-confirmed microRNA-disease associations and introducing the expression profile data of microRNAs for the first time. Compared with the microRNA functional similarity and microRNA Gaussian interaction profile kernel similarity which have been widely used by previously-proposed prediction models, as the information resource of microRNA expression similarity, the expression profiles of microRNA is directly collected from the biological experiments and therefore causes less prediction error and bias for the prediction. The excellent prediction performance of EPMDA has been demonstrated by the cross validation experiments, case studies and the comparison with some classical algorithms and existing prediction models. It could be anticipated that EPMDA can be used as a useful tool for further biological researches and drug developments.

The reasons of good performance of EPMDA may come from the following factors. Firstly, EPMDA is the first computational model which introduces microRNA expression profiles as inputs. Compared with the other kinds of microRNA similarity, the similarity of microRNA expression level can be directly computed by the experimental data and therefore is more reliable for predicting microRNA-disease associations. Specially, it should be noted that the expression profiles of some microRNAs are still unavailable partially because the database of microRNA.org has not been updated for a long time. We anticipate that EPMDA can achieve better prediction performance with more complete information resource in the future. In addition, the basic assumption of EPMDA that microRNAs sharing similar distribution in different human tissues and cell lines tend to be involved in similar disease is reasonable and feasible for the problem of microRNA-disease association prediction. Finally, the two-way diffusion method proposed in this work and the kind of input data fit well together, which has been demonstrated by the comparison with other recommendation algorithms and social network prediction algorithm. It is effective to use the proposed method to retain the important information based on the known microRNA-disease association network which can be regarded as a classical bipartite graph. However, there are also some limitations existing in the model of EPMDA. For example, the EPMDA cannot be applied to the new disease which has no record of associated microRNAs. Besides, the problem of selecting parameter values is still not well solved since the damping coefficients were roughly set as 0.5 in this work.

## MATERIALS AND METHODS

The dataset explored in this work was downloaded from HMDD v2.0 database (http://www.cuilab.cn/hmdd). The current version of HMDD has collected 10368 entries covering 572 microRNA genes and 378 kinds of disease from 3511 papers. We downloaded the microRNA-disease association from HMDD and removed the repetitive records[26]. The final explored dataset consists of 5430 known microRNA-disease associations covering 495 microRNAs and 383 diseases. To obtain the information of expression distribution of microRNAs, we downloaded the expression profile data from the latest released version of microRNA.org database (http://www.microrna.org/microrna/home.do). In each record of microrna.org database, the expression level of microRNAs in 172 human tissues and cell lines is recorded [49]. As a result, we obtained the expression profile data of 315 microRNAs which are recorded in HMDD database.

### MicroRNA expression similarity

Based on the assumption that microRNAs which share similar expression distribution in human tissues are more possible to get involved in the mechanism of similar disease, we first proposed a microRNA similarity measure for predicting microRNA-disease associations. Specifically, all microRNAs were represented by 172-dimension vectors which record the expression level in 172 human tissues and cell lines. In this work, the expression profile data of 315 out of 495 microRNAs recorded in HMDD database were collected from microrna.org database. We further adopted the Pearson correlation coefficient to measure the expression similarity of each microRNA pairs. Given the expression profiles of two microRNA (say *e_mi_* and *e_mj_*), we calculated their similarity as follow:

$$r(e_{m_{i}},e_{m_{j}})=\frac{\sum \left(e_{m_{i}}-\bar{e_{m_{i}}})(e_{m_{j}}-\bar{e_{m_{j}}}\right)}{\sqrt{{\sum \left(e_{m_{i}}-\bar{e_{m_{i}}}\right)}^{2}{\sum \left(e_{m_{j}}-\bar{e_{m_{j}}}\right)}^{2}}}$$ (1)

where $\bar{e_{m_{i}}}$ and $\bar{e_{m_{j}}}$ denote the means of vector *e_mi_* and *e_mj_*, respectively. For those microRNA-microRNA pairs in which one microRNA expression profile is unavailable, we simply set its similarity as the mean of the similarities of rest computable pairs. As a result, we constructed a microRNA expression similarity matrix *SM_microRNA_* in which entity *SM_microRNA_(i,j)* is the computed expression similarity between microRNA *m_i_* and *m_j_*. To further evaluate the prediction results of EPMDA in the case study section, we also explored two other databases (i.e., miR2Disease and dbDEMC) which totally store 3273 and 2224 microRNA-disease associations, respectively.

### Disease semantic similarity

Mesh database (http://www.ncbi.nlm.nih.gov/) offer a comprehensive annotation for diverse human complex disease, which help researchers to study the relationship among different diseases from different perspectives [50]. In this work, we simply adopted the popular disease semantic similarity measure which has been widely used in previous works [37–41]. Specifically, the features of diseases were represented by the corresponding Directed Acyclic Graph (DAG) composed of disease Mesh descriptors. As the first step to calculate disease semantic similarity, we computed the semantic contribution of each DAG term (say *t*) to the disease *d* based on its DAG *D* as follow:

$$\{\begin{array}{cc} D_{d}(t)=1 & \text{if }t=d\\ D_{d}(t)=\max\{\Delta *D_{d}(t')|t'\in C(d)\}\text{if }t\neq d & \end{array}$$ (2)

where *C(d)* is the set of children terms of disease term *d* in DAG *D*; Δ denotes the semantic contribution decay factor. Following previous works, we set Δ to be 0.5. For each disease pair (say *d_i_-d_j_*), we calculated their semantic similarity score *SS(d_i_,d_j_)* as follow:

$$SM_{\text{disease}}(d_{i},d_{j})=\frac{\sum_{t\in (A(d_{i})\cap A(d_{j}))}\left(D_{d_{i}}(t)+D_{d_{j}}(t)\right)}{\sum_{t\in A(d_{i})}D_{d_{i}}(t)+\sum_{t\in A(d_{j})}D_{d_{j}}(t)}$$ (3)

where *A(d_i_)* and *A(d_j_)* denote the sets of ancestor nodes of disease term *d_i_* and *d_j_*, respectively. As a result, we finally obtained a disease similarity matrix *SM_disease_* whose size was 378×378.

### EPMDA

**Figure 3:**
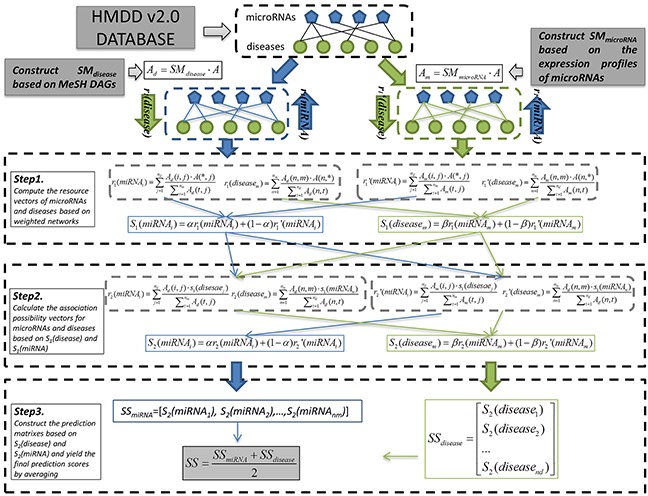
Flowchart of computational process of EPMDA based on the disease semantic similarity and microRNA expression similarity

Based on the assumption that microRNAs which have similarity expression distribution in human tissues tend to get involved in similar diseases, we developed EPMDA which is the first computational model introducing expression profile data of microRNAs for microRNA-disease association prediction. Specifically, EPMDA applies a two-way diffusion algorithm to calculate the association possibility of each microRNA-disease pair by combing microRNA expression similarity, disease semantic similarity, and known microRNA-disease associations (see Figure 3). Specifically, the information resource of microRNA and disease nodes flow back and forth between each other by two steps based on the bipartite graph. In the stage of data preprocessing, EPMDA first computes two similarity matrixes (i.e. microRNA similarity matrix and disease similarity matrix) based on MeSH DAGs and microRNA expression profiles, and then constructs two weighted microRNA-disease association networks with corresponding adjacency matrixes, *A_d_* and *A_m_*, respectively:

$$A_{d}=SM_{\text{disease}}\cdot A$$ (4)

$$A_{m}=SM_{\text{microRNA}}\cdot A$$ (5)

where *A* is the adjacency matrix of the known microRNA-disease association network recorded in HMDD v2.0 database. There are three main steps for prediction computing in the model of EPMDA. In the first step, we computed the resource vectors for microRNA and disease nodes based on disease-based weighted network as follows:

$$r_{1}({\text{miRNA}}_{i})=\sum_{j=1}^{n_{d}}\frac{A_{d}(i,j)\cdot A(*,j)}{\sum_{t=1}^{n_{m}}A_{d}(t,j)}$$ (6)

$$r_{1}({\text{disease}}_{m})=\sum_{n=1}^{n_{m}}\frac{A_{d}(n,m)\cdot A(n,*)}{\sum_{t=1}^{n_{d}}A_{d}(n,t)}$$ (7)

where A(^*^,j) denote the j-th row vector in matrix *A*; *n_d_* is the number of diseases; *n_m_* is the number of microRNAs. The resource vector *r_1_(miRNA_i_)* which is a *n_d_*-dimension column vector describes the weights diffusing from all disease nodes to the node of i-th microRNA. Similarly, the row vector of *r_1_(disease_m_)* describes the weights diffusing from all microRNA nodes to the node of *m-th* disease. Based on the microRNA-based weight network, we computed the resource vectors for miRNA and disease nodes in a similar way:

r1'(miRNAi)=∑j=1ndAm(i,j)⋅A(*,j)∑t=1nmAm(t,j) (8)

r1'(diseasem)=∑n=1nmAm(n,m)⋅A(n,*)∑t=1ndAm(n,t) (9)

Then we computed the integrated resource vectors of microRNAs and diseases based on the two weighted networks:

S1(miRNAi)=αr1(miRNAi)+(1−α)r1'(miRNAi) (10)

S1(diseasem)=βr1(miRNAm)+(1−β)r1'(miRNAm) (11)

where *α* and *β* is damping coefficient for balancing the contribution between disease-based weighted network and microRNA-based weighted network. In this work, we simply set α and β as 0.5. In the second step, EPMDA model calculates feedback resource vectors for microRNAs and diseases based on disease-based network and the computed *S_1_(miRNA_i_)* and *S_1_(disease_m_)* as follows:

$$r_{2}({\text{miRNA}}_{i})=\sum_{j=1}^{n_{d}}\frac{A_{d}(i,j)\cdot s_{1}({\text{disesae}}_{j})}{\sum_{t=1}^{n_{m}}A_{d}(t,j)}$$ (12)

$$r_{2}({\text{disease}}_{m})=\sum_{n=1}^{n_{m}}\frac{A_{d}(n,m)\cdot s_{1}({\text{miRNA}}_{n})}{\sum_{t=1}^{n_{d}}A_{d}(n,t)}$$ (13)

Similarly, the feedback resource vectors for microRNAs and diseases were also computed based on the microRNA-based weighted network:

r2'(miRNAi)=∑j=1ndAm(i,j)⋅s1(disesaej)∑t=1nmAm(t,j) (14)

r2'(diseasem)=∑n=1nmAd(n,m)⋅s1(miRNAn)∑t=1ndAd(n,t) (15)

We can further obtain final prediction scores by combining the feedback resource vectors:

S2(miRNAi)=αr2(miRNAi)+(1−α)r2'(miRNAi) (16)

S2(diseasem)=βr2(miRNAm)+(1−β)r2'(miRNAm) (17)

Clearly, *S_2_(miRNA_i_)* is a *n_d_*-dimension column vector which describes prediction scores for *n_d_* diseases to be associated with *i*-th microRNA, and *S_2_(disease_m_)* describes to possibility of *n_m_* microRNAs to be associated with *m*-th disease. In the third step, EPMDA constructs two prediction matrixes, *SS_miRNA_* and *SS_disease_*, by simply concatenating two kinds of feedback resource vectors of microRNAs and diseases:

$$SS_{\text{miRNA}}=[S_{2}({\text{miRNA}}_{1}),\;S_{2}({\text{miRNA}}_{2}),\ldots ,S_{2}({\text{miRNA}}_{n_{m}})]$$ (18)

$$SS_{\text{disease}}={\left[S_{2}{\left({\text{disease}}_{1}\right)}^{T},\;S_{2}{\left({\text{miRNA}}_{2}\right)}^{T},\ldots ,S_{2}{\left({\text{miRNA}}_{n_{m}}\right)}^{T}\right]}^{T}$$ (19)

Finally, the final prediction matrix SS yielded by EPMDA model is computed by simply averaging *SS_miRNA_* and *SS_disease_*:

$$\text{SS}=\frac{SS_{\text{miRNA}}+SS_{\text{disease}}}{2}$$ (20)

where the entity *SS(i,j)* of matrix *SS* denote the predicted association possibility for *i*-th microRNA to be associated with *j*-th disease.

## SUPPLEMENTARY MATERIALS TABLE





## References

[1] Lu J, Getz G, Miska EA, Alvarez-Saavedra E, Lamb J, Peck D, Sweet-Cordero A, Ebert BL, Mak RH, Ferrando AA (2005). MicroRNA expression profiles classify human cancers. nature.

[2] Griffiths-Jones S, Grocock RJ, van Dongen S, Bateman A, Enright AJ (2006). miRBase: microRNA sequences, targets and gene nomenclature. Nucleic Acids Res.

[3] Calin GA, Croce CM (2006). MicroRNA signatures in human cancers. Nat Rev Cancer.

[4] Iorio MV, Ferracin M, Liu CG, Veronese A, Spizzo R, Sabbioni S, Magri E, Pedriali M, Fabbri M, Campiglio M, Ménard S, Palazzo JP, Rosenberg A (2005). MicroRNA gene expression deregulation in human breast cancer. Cancer Res.

[5] He L, Thomson JM, Hemann MT, Hernando-Monge E, Mu D, Goodson S, Powers S, Cordon-Cardo C, Lowe SW, Hannon GJ, Hammond SM (2005). A microRNA polycistron as a potential human oncogene. Nature.

[6] Kozomara A, Griffiths-Jones S (2011). miRBase: integrating microRNA annotation and deep-sequencing data. Nucleic Acids Res.

[7] Consortium EP, ENCODE Project Consortium (2004). The ENCODE (ENCyclopedia of DNA elements) project. Science.

[8] Birney E, Stamatoyannopoulos JA, Dutta A, Guigó R, Gingeras TR, Margulies EH, Weng Z, Snyder M, Dermitzakis ET, Thurman RE, Kuehn MS, Taylor CM, Neph S, Children's Hospital Oakland Research Institute (2007). Identification and analysis of functional elements in 1% of the human genome by the ENCODE pilot project. Nature.

[9] Lee RC, Feinbaum RL, Ambros V. (1993). The C. elegans heterochronic gene lin-4 encodes small RNAs with antisense complementarity to lin-14. Cell.

[10] Reinhart BJ, Slack FJ, Basson M, Pasquinelli AE, Bettinger JC, Rougvie AE, Horvitz HR, Ruvkun G (2000). The 21-nucleotide let-7 RNA regulates developmental timing in Caenorhabditis elegans. Nature.

[11] Salmena L, Poliseno L, Tay Y, Kats L, Pandolfi PP (2011). A ceRNA hypothesis: the Rosetta Stone of a hidden RNA language?. Cell.

[12] Cesana M, Cacchiarelli D, Legnini I, Santini T, Sthandier O, Chinappi M, Tramontano A, Bozzoni I (2011). A long noncoding RNA controls muscle differentiation by functioning as a competing endogenous RNA. Cell.

[13] Sumazin P, Yang X, Chiu HS, Chung WJ, Iyer A, Llobet-Navas D, Rajbhandari P, Bansal M, Guarnieri P, Silva J, Califano A (2011). An extensive microRNA-mediated network of RNA-RNA interactions regulates established oncogenic pathways in glioblastoma. Cell.

[14] Karreth FA, Tay Y, Perna D, Ala U, Tan SM, Rust AG, DeNicola G, Webster KA, Weiss D, Perez-Mancera PA, Krauthammer M, Halaban R, Provero P (2011). In vivo identification of tumor- suppressive PTEN ceRNAs in an oncogenic BRAF-induced mouse model of melanoma. Cell.

[15] Poliseno L, Pandolfi PP (2015). PTEN ceRNA networks in human cancer. Methods.

[16] Alshalalfa M, Alhajj R (2013). Using context-specific effect of miRNAs to identify functional associations between miRNAs and gene signatures. BMC Bioinformatics.

[17] Bartel DP (2009). MicroRNAs: target recognition and regulatory functions. Cell.

[18] Miska EA (2005). How microRNAs control cell division, differentiation and death. Curr Opin Genet Dev.

[19] Cheng AM, Byrom MW, Shelton J, Ford LP (2005). Antisense inhibition of human miRNAs and indications for an involvement of miRNA in cell growth and apoptosis. Nucleic Acids Res.

[20] Cui Q, Yu Z, Purisima EO, Wang E (2006). Principles of microRNA regulation of a human cellular signaling network. Mol Syst Biol.

[21] Xu P, Guo M, Hay BA (2004). MicroRNAs and the regulation of cell death. Trends Genet.

[22] Goh JN, Loo SY, Datta A, Siveen KS, Yap WN, Cai W, Shin EM, Wang C, Kim JE, Chan M (2016). microRNAs in breast cancer: regulatory roles governing the hallmarks of cancer. Biol Rev Camb Philos Soc.

[23] Cho WC, Chow AS, Au JS (2011). MiR-145 inhibits cell proliferation of human lung adenocarcinoma by targeting EGFR and NUDT1. RNA Biol.

[24] Vickers MM, Bar J, Gorn-Hondermann I, Yarom N, Daneshmand M, Hanson JE, Addison CL, Asmis TR, Jonker DJ, Maroun J, Lorimer IA, Goss GD, Dimitroulakos J (2012). Stage-dependent differential expression of microRNAs in colorectal cancer: potential role as markers of metastatic disease. Clin Exp Metastasis.

[25] Jiang Q, Wang Y, Hao Y, Juan L, Teng M, Zhang X, Li M, Wang G, Liu Y (2009). miR2Disease: a manually curated database for microRNA deregulation in human disease. Nucleic Acids Res.

[26] Li Y, Qiu C, Tu J, Geng B, Yang J, Jiang T, Cui Q (2014). HMDD v2.0: a database for experimentally supported human microRNA and disease associations. Nucleic Acids Res.

[27] Yang Z, Ren F, Liu C, He S, Sun G, Gao Q, Yao L, Zhang Y, Miao R, Cao Y, Zhao Y, Zhong Y, Zhao H (2010). dbDEMC: a database of differentially expressed miRNAs in human cancers. BMC Genomics.

[28] Xuan P, Han K, Guo Y, Li J, Li X, Zhong Y, Zhang Z, Ding J (2015). Prediction of potential disease-associated microRNAs based on random walk. Bioinformatics.

[29] Xu J, Li CX, Lv JY, Li YS, Xiao Y, Shao TT, Huo X, Li X, Zou Y, Han QL, Li X, Wang LH, Ren H (2011). Prioritizing candidate disease miRNAs by topological features in the miRNA target-dysregulated network: case study of prostate cancer. Mol Cancer Ther.

[30] Mørk S, Pletscher-Frankild S, Caro AP, Gorodkin J, Jensen LJ (2014). Protein-driven inference of miRNA-disease associations. Bioinformatics.

[31] Murakami Y, Yasuda T, Saigo K, Urashima T, Toyoda H, Okanoue T, Shimotohno K (2006). Comprehensive analysis of microRNA expression patterns in hepatocellular carcinoma and non-tumorous tissues. Oncogene.

[32] Ikeda S, Kong SW, Lu J, Bisping E, Zhang H, Allen PD, Golub TR, Pieske B, Pu WT (2007). Altered microRNA expression in human heart disease. Physiol Genomics.

[33] Kloosterman WP, Plasterk RH (2006). The diverse functions of microRNAs in animal development and disease. Dev Cell.

[34] Li JQ, Rong ZH, Chen X, Yan GY, You ZH (2017). MCMDA: matrix completion for MiRNA-disease association prediction. Oncotarget.

[35] Chen X, Huang YA, You ZH, Yan GY, Wang XS (2017). A novel approach based on KATZ measure to predict associations of human microbiota with non-infectious diseases. Bioinformatics.

[36] Chen X (2015). KATZLDA: KATZ measure for the lncRNA-disease association prediction. Sci Rep.

[37] Chen X, Yan CC, Zhang X, You ZH, Deng L, Liu Y, Zhang Y, Dai Q (2016). WBSMDA: within and between score for MiRNA-disease association prediction. Sci Rep.

[38] Chen X, Yan GY (2014). Semi-supervised learning for potential human microRNA-disease associations inference. Sci Rep.

[39] Chen X, Liu MX, Yan GY (2012). RWRMDA: predicting novel human microRNA-disease associations. Mol Biosyst.

[40] Chen X, Yan CC, Zhang X, Li Z, Deng L, Zhang Y, Dai Q (2015). RBMMMDA: predicting multiple types of disease-microRNA associations. Sci Rep.

[41] Chen X, Yan CC, Zhang X, You ZH, Huang YA, Yan GY (2016). HGIMDA: heterogeneous graph inference for miRNA-disease association prediction. Oncotarget.

[42] Xuan P, Han K, Guo M, Guo Y, Li J, Ding J, Liu Y, Dai Q, Li J, Teng Z, Huang Y (2013). Prediction of microRNAs associated with human diseases based on weighted k most similar neighbors. PLoS One.

[43] Wang D, Wang J, Lu M, Song F, Cui Q (2010). Inferring the human microRNA functional similarity and functional network based on microRNA-associated diseases. Bioinformatics.

[44] Jemal A, Bray F, Center MM, Ferlay J, Ward E, Forman D (2011). Global cancer statistics. CA Cancer J Clin.

[45] Drusco A, Nuovo GJ, Zanesi N, Di Leva G, Pichiorri F, Volinia S, Fernandez C, Antenucci A, Costinean S, Bottoni A, Rosito IA, Liu CG, Burch A (2014). MicroRNA profiles discriminate among colon cancer metastasis. PLoS One.

[46] Saini S, Arora S, Majid S, Hirata H, Dahiya R (2014). MicroRNAs in the Development and Progression of Kidney Cancer. MicroRNA in Development and in the Progression of Cancer.

[47] Gottardo F, Liu CG, Ferracin M, Calin GA, Fassan M, Bassi P, Sevignani C, Byrne D, Negrini M, Pagano F (2007). Micro-RNA profiling in kidney and bladder cancers. Urol Oncol.

[48] Wulfken LM, Moritz R, Ohlmann C, Holdenrieder S, Jung V, Becker F, Herrmann E, Walgenbach-Brünagel G, von Ruecker A, Müller SC, Ellinger J (2011). MicroRNAs in renal cell carcinoma: diagnostic implications of serum miR-1233 levels. PLoS One.

[49] Betel D, Wilson M, Gabow A, Marks DS, Sander C (2008). The microRNA.org resource: targets and expression. Nucleic Acids Res.

[50] Lipscomb CE (2000). Medical subject headings (MeSH). Bull Med Libr Assoc.

