# Supplementary material for: EPMDA: an expression-profile based computational model for microRNA-disease association prediction
Source: Oncotarget. 2017 Jun 28;8(50):87033–43. doi: 10.18632/oncotarget.18788 (PMC5675613; doi:10.18632/oncotarget.18788)
Supplement: Supplementary file 1 [file oncotarget-08-87033-s001.pdf]

EPMDA: an expression-profile based computational model for microRNA-disease association prediction

SUPPLEMENTARY MATERIALS

Supplementary Table 1: The rank list of most potential microRNA-disease associations predicted by EPMDA based on HMDD database (XLSX).

See Supplementary File 1
